# A systematic managerial perspective on the environmentally sustainable construction practices of UK

**DOI:** 10.1007/s11356-022-20255-5

**Published:** 2022-04-26

**Authors:** Rashid Maqbool, Ifeanyi Echezona Amaechi

**Affiliations:** Department of Mechanical and Construction Engineering, Northumbria University, Newcastle upon Tyne, NE1 8ST UK

**Keywords:** Sustainable construction, Sustainable development, Managerial perspective, Barriers, UK

## Abstract

Construction industry, though is the backbone of any economy, still add a significant portion of emissions, utilising energy supplies, and reasoning in bulk of waste production. The sustainable construction practices are the only solution considering the global climatic challenges. Owing its enormous benefits, a lot of sustainable constructions projects are built around the world, both in developed and developing countries. However, considering the innovative material and technological involvement, and lack of knowledge and expertise, such sustainable construction projects are not always successful. This research aims to investigate the barriers and factors impacting sustainability in the construction projects. More specifically, its primary purpose is to have the perspective of managers on the actors and barriers of sustainable construction in the UK. A mixed method was used to collect the data, one in the mean of questionnaire survey, and the second through the case study. To acquire quantitative data, a snowball sampling was applied to collect the questionnaire survey based data from 128 UK construction managerial positions, such as system managers, sustainability managers, project managers and construction managers, etc. The quantitative acquired data was analysed using mean analysis, relevant importance index (RII), correlation and multiple hierarchical regression. The RII analysis discovered that sustainable construction designs is a top drivers of sustainable construction practices, whereas excessive concentration on price is found as the top impediment of sustainable construction practices. It was also shown by the hierarchical regression analysis that stakeholders factors, project management factors and technological factors significantly impact to sustainable construction practice. However, surprisingly the role of barriers was not observed in the sustainable construction practices of the UK. The same findings were also confirmed with the case study analysis of the Kier Group plc, which believes in the sustainable construction practices. Hence, it is needful for the positive sides of these factors be considered and duly exploited. The research findings provide interesting industrial insights towards sustainable construction projects, while providing useful directions to the industrialists, policymakers and construction professionals, not only by reducing the unfavourable effects, but also by proposing the intention of restoring factors of the environment, economic and social sustainability.

## Introduction

Sustainable options are vital to bring into the construction industry these days, and experts and policymakers are seemingly keen to focus on the relevant strategies, policies and practices to convert the construction practices on sustainable path (Maqbool et al. 2020a, b). On the same angle, the UK, being a developed country, has its sustainable and environment friendly progressions in the construction industry, with different policies, legislation and modern methods of construction (Akadiri and Fadiya 2013). Alongside this industry is equally concerned with the usage of the sustainable resources management and providing the high-quality sustainable construction projects.

Whilst the UK construction industry has seen significant expansion and has taken positive steps to encourage sustainable construction, several roadblocks have emerged in the form of legislative restrictions and a lack of technical skills (Chan et al. 2017). The building industry’s success is being hampered by a lack of understanding among clients and customers about sustainable construction approaches (Djokoto et al. 2014). Even though the UK construction sector is developing eco-friendly ways, ideas and innovation to encourage sustainable construction, a lack of demand from clients is causing problems (Ohiomah et al. 2019).

Many aspects have been discovered to be important in achieving sustainable construction. Some studies have looked at the long-term viability of building materials (Häkkinen and Belloni 2011), others have looked at the long-term viability of the supervisory process (Huovila and Koskela 1998), while still others have looked at it from an economic standpoint (Gunduz and Almuajebh 2020). Most construction businesses have a project team, which is usually led by a project or construction manager, and whether the project team’s leadership competence results in a long-term construction is a critical question (Maqbool et al. 2017). This study was prompted by the large gap in knowledge surrounding the problem of sustainability. Every sector has recognised stakeholders whose decisions, efforts, and policies have an impact on the industry’s overall goal (Maqbool et al. 2020c). Policy, culture, value system, and direction in reaching industrial goals are all shaped and charted by industry specialists, leadership, and other stakeholders. When we realise that every construction project is driven by human beings, we grasp the importance of experts with the knowledge, skills, leadership qualities, and attitude to coordinate project sustainability (Lam et al. 2010). This research study examines the barriers and problems of sustainable construction from a managerial perspective in the context of the UK construction industry. The link between managerial ability and long-term construction is still a source of worry. As a result, the goal of this research is to look at the impediments and drivers to sustainable construction from a managerial standpoint.

A lot of factors play important role in the sustainable construction projects; however, the role of project manager is seemed to be the most critical one among all. A construction project manager follows certain standards, industrial codes, professional ethics, settled policies and own skills, knowledge and expertise while delivering the sustainable construction projects (Delnavaz 2012). Besides, the role of individual organisations and policy departments found to be important for delivering such important and sustainable construction projects, which help in developing a new market of sustainable development and effective projects. A study conducted by Marichova (2020) provided the important role of government’s relevant actions and effective strategies to pave way for the stakeholders to provide quality results in the construction industry, which lead a sustainable development. Similarly, Opoku et al. (2015), found the role of organisational leadership in engaging the internal and external stakeholders for keeping a sustainable construction intention in the UK. This study covers some of important sustainable practices for the UK construction industry; however, the suggestions are mostly limited to the role of leadership in the construction firms. The major difference is that this study does not provide any suggestion regarding the barriers and actions of the modern sustainable construction, where the technological changes are constant and global warming issues are prevailing with the passage of time. Considering this all, there is a dire need of any research to provide detailed managerial overview about the current industrial barriers hindering the sustainable practices, and providing the sustainable actions. Owing this an important research gap can be filled by shaping the role of sustainable construction in these uncertain situations and keeping an eye on the most important aspect of climate changes.

This research aimed to investigate the prevailing barriers and possible factors impacting sustainability in construction industry in the UK. In order to attain the aim, the following objectives are designed to test in this study:

i. Determining actions required to stimulate sustainable construction in the UK
ii. Examining the barriers to adopting the best practice and policy for sustainable construction in the country
iii. Examining the relationship between barriers, factors impacting sustainability in sustainable construction in the UK

**Fig. 1:**
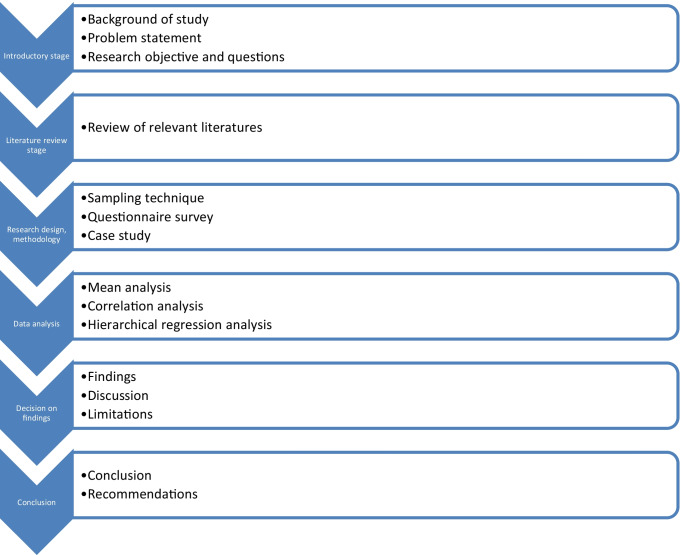
Research flowchart

This research is designed to flow in six stages. which encompass the following Fig. 1.

This study is significant in contributing to the already existing literature on sustainable construction in the UK and the world at large. Besides, the study is also important, as it helps in providing the state of the art on the barriers on construction and possible factors impacting the sustainability in the construction industry of the UK. The findings of this study are expected to equally important for the policymakers for planning and bringing better legislations for industry, for investors to decide their intentions for better sustainability and high return on investments, and for construction professionals who are ready to adopt modern methods for utilising their expertise to shape a better sustainable future. It is worth mentioning that the findings of this study would be also important for the researchers to have a way for producing quality research direction to bring innovative solutions for different communities across the world.

## Literature review

### Sustainable development in the UK

**Fig. 2:**
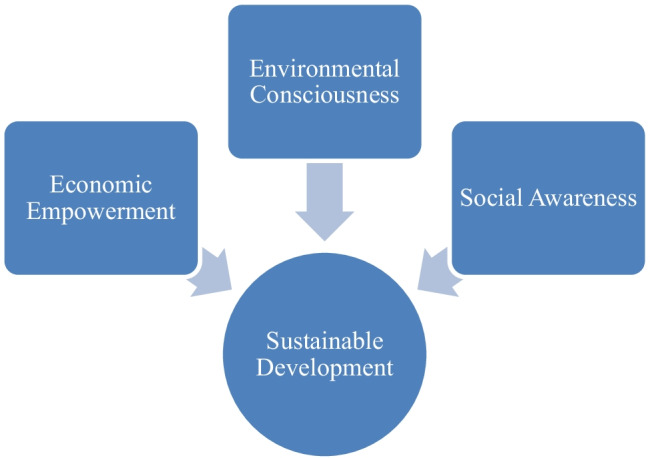
Three pillars of sustainable development

Sustainability in human activities and sustainable development of productions is a serious issue. This ensures cleaner environment, less pollutants in the atmosphere and water bodies, preservation of forests to conserve endangered plants and animals. Sustainable development is undeniably attractive, and it has the potential to make the world a better and healthier place for everyone (Maqbool and Wood 2022; Maqbool et al. 2018). However, most countries are still striving to translate this concept into a concrete and visible term (Cotgrave and Riley 2013). Opoku (2019) suggested that among the three pillars of sustainable development, the social justice aspect of sustainable construction is the most challenging to address in individual projects. In this regard, Baldwin (2013) argues that most discussions about sustainability have mostly served as a forum for expressing emotions and views, with no rigorous analysis of sustainability or sustainable paths for the modern industrial economy. The three pillars of sustainability are diagrammatically represented in Fig. 2.

### Construction industry and sustainable development in the UK

The United Nations Conference on Environment and Development’s Earth Summit of 1992 produced an action plan called Agenda 21 which outlined 27 principles of sustainable development (United Nations (UN) 1992)). Based on the mandate, one of the first countries to develop a sustainable development strategy was the UK in 1994 (Pitt et al. 2009). One of the UK targets was to cut down on emissions of greenhouse gas (Gunatilake 2013). Following this was a published document in year 2000 for sustainable construction in the UK by Department of the Environment Transport and Regions (Gunatilake 2013). The sustainable construction strategy specified ways the construction industry can contribute to sustainable development like developing quality houses that improve health and wellbeing of residents, reduction in energy use, conservation and preservation of natural resources and ecosystem, and lastly to be more competitive and financially rewarding (DETR 2000).

The significance of activities of construction to attaining sustainable development is huge and cannot be disputed (Gunatilake 2013). In the UK, it costs about 40–50% of entire country’s energy use to construct, operate and for final deconstruction of buildings (Williams and Dair 2007; Garde 2009). This type of impact is witnessed and recorded by researchers in the number of materials exploited for construction. According to Sev (2009), about 380 million tonnes of natural resources and raw materials are used every year by the UK construction industry. In addition, there is almost 13 million tonnes and 100 million tonnes of unused materials and waste produced each year respectively (Garde 2009). Sustainable development in construction industry can be achieved when everyone is involved and committed to cut down on negative ecological and socio-economic impacts (Parkin 2000; Parkin, et al. 2003).

In the UK construction industry, the interest on sustainable construction is increasingly on the rise (Choguill 2008). This is because of research and improvements in technology, awareness campaign from non-governmental bodies and policies from relevant government authorities (Hwang and Tan 2012). However, there seems to be some hinderances to full and complete compliance to the principles of sustainable development in sustainable construction concept (Choguill 2008). These challenges emanate in different dimensions and sources causing barriers to widespread adoption of sustainable construction (AlSanad 2015). The sources could be from government’s inactions, stakeholders’ poor understanding, human resources, economic implications and culture (Son et al. 2011). There are barriers as client’s un-interestedness or unawareness, huge cost or inadequate information on long-time financial benefits, lack of sustainable materials, poor or lack of rules and regulations, and slow adoption of integrated modern methods of construction (Bond 2011a, 2011b). Despite government set regulations, researchers’ efforts, and advancement in technology and innovations, these barriers have made sustainable construction unpopular and contributed to the low demand (Zhou and Lowe 2003).

### Factors impacting on sustainable construction

#### Stakeholders roles

Stakeholders contribute to either the barriers or actions of sustainable construction depending on the circumstances (Maqbool 2018). It becomes a barrier where a potential owner shows no interest or support to adopting principles of sustainable development (Samari et al. 2013; Zhang et al. 2015; Toor and Ofori 2008; Zhang 2014). This challenge is mostly in connection with stakeholders limited understanding of what sustainability entails (Pitt et al. 2009; Serpell et al. 2013). This is related to poor knowledge on the financial and social benefits of sustainable construction known to stakeholders (Zhang et al. 2013), having low level knowledge of innovation in sustainability (Ahn et al. 2013) and failure to build collaborative working environment amongst stakeholders (Richardson and Lynes 2007).

#### Project management practices

According to Reffat (2004), another important concern facing the construction sector in terms of professional skills is a lack of human resource capacity. The implementation of government policy initiatives, according to a 1999 CIB analysis, necessitates the utilisation of persons with managerial abilities (Raynsford 1999). Professionals in the construction sector are expected to be well-versed in the working principles of sustainable building so that they can apply sustainable policies in practice. According to Nguyen et al. (2017), any industry’s personnel is its backbone; thus, individuals who are not only knowledgeable but also can support sustainable construction while working as a team are needed.

In another vein, organisations have shown quality project management practices in project teams that drives sustainability in construction through team commitment (Quinn and Dalton 2009), policy implementation efforts (Gattiker and Carter 2009), realisation of incentive policy (Avery 2005), project team skills (Opoku et al. 2015), sustainable procurement model (Toor and Ofori 2008), commit to changing behaviour (Holton et al. 2008) and appropriate project organisation structure (Northouse 2021).

#### Technological factors

There has been a significant investment and study in technologically innovative ideas that has proven to enhance construction industry’s agenda towards promoting sustainable development principles in construction (Maqbool and Sudong 2018). The UK construction sector has largely continued to witness technological advancements and innovative inputs in form of development as well as application of BIM technologies, implementation of modern methods of construction like off-site fabrications and utilisation of reusable materials, and Lean methodology.

### Barriers of sustainable construction practices in the UK

Despite a plethora of policies and guidelines, the construction sector nevertheless faces several obstacles that prevent sustainable development from being fully realised in practice (Brennan and Cotgrave 2014). Many roadblocks and challenges have remained in the way of total acceptance of sustainable development in the construction sector around the world (Balo 2003; Dalibi et al. 2017; Gan et al. 2015). Even when attempts are made to promulgate regulative agendas and regulations to guide on sustainability in construction industry practices, the construction sector in the UK continues to encounter these challenges (Sourani and Sohail 2011; Williams and Dair 2007).

#### Economic impacts

A lack of understanding of the economic benefits of sustainable construction contributes to a slew of roadblocks to the practice (Daniel et al. 2018). From an economic standpoint, cost has been identified as a major impediment to sustainable construction, as several academics have pointed out (Sodagar and Fieldson 2008; Häkkinen and Belloni 2011). Many owners cannot afford or are unwilling to pay for the high prices of materials, technologies and knowledge connected with sustainable construction. Knowing that clients pay for projects and decide where the funds should be spent, this becomes a barrier for sustainable construction drives (Zhou and Lowe 2003). Excessive focus and attention on a project’s expenses in sustainable construction could jeopardise the adoption of sustainable practices that are required to make such construction green and sustainable (Häkkinen and Belloni 2011). Summary of some of the important studies on the contributions of economy towards sustainable construction is presented in Table 1.

**Table 1:** Contributions of the economy to barriers of SC

Factors	Sources	Methodology
Poor understanding of the economic benefits	Zhou and Lowe (2003)	Literature review
Excessive concentration and attention to the costs	Häkkinen and Belloni (2011)	Case study, literature review, interview
Potential delay in schedule or abandonment	Hayles and Kooloos, (2008); Richardson and Lynes (2007)	Case study, semi‐structured in‐depth interviews
Higher costs in materials, technologies, and expertise	Safinia et al. (2017)	Literature review

#### Cultural limitations

The resistance of valuable and tangible new ideas and changes in construction industry whilst retaining current practices is a barrier (Williams and Dair 2007). Ametepey et al. (2015) observed that the culture of rejecting adoption of sustainable development in construction is a drawback to sustainable construction. Hwang and Tan (2012) attributed it to be either they lack necessary skills or outright disregard to take on new initiatives and innovation when presented. Opoku and Ahmed (2014) agreed with the assertion that a barrier to sustainable construction could come from the inefficiency of project managers, they went further to state that lack of human resources equally poses a hinderance. Again, Hwang and Tan (2012) observed that nonchalant attitude and poor cooperation of project team members to collaborate on sustainability drive makes it difficult to attain sustainable construction. Summary of some of the important studies on the role of culture towards sustainable construction is presented in Table 2.

**Table 2:** Roles of culture to barriers of SC

Factors	Sources	Methodology
Resisting valuable and tangible new ideas and changes	Williams and Dair (2007); Ametepey et al., (2015)	Case studies
Poor cooperation of project team members	Hwang and Tan (2012)	Literature review, survey questionnaire

#### Government roles

Government laws and legislation are one of the most important drivers of sustainable building, but a lack of applicable rules and policies is one of the most significant impediments or challenges to achieving absolute sustainability in the construction industry (Heeres et al. 2004). Serpell et al. (2013) and Samari et al. (2013) both stated in their studies that the lack of government legislation providing rules to require construction sector players on sustainability is a major setback. Government incentives to help organisations’ sustainability efforts are occasionally lacking. These incentives could take the form of monetary rewards or tax exemptions for every action taken to reduce environmental and social impacts such as noise pollution, gas emissions and solid waste (Chang et al. 2016; Chen et al. 2015; Samari et al. 2013; Shi et al. 2016; Zhang et al. 2015). Summary of some of the important studies on the government contributions towards sustainable construction is presented in Table 3.

**Table 3:** Government contributions to barriers of SC

Factors	Sources	Methodology
Government regulations and legislation	Heeres et al. (2004); Serpell et al. (2013); Samari et al. (2013)	Questionnaire survey, case study, literature review
Lack of incentives from government	Zhang et al. (2015); Samari et al. (2013); Zhang et al. (2013); Chen et al. (2015); Chang et al. (2016); Shi et al. (2016)	Survey questionnaire, semi-structured interviews, case study

#### Resources factors

Another impediment to sustainable construction is a lack of sustainable materials and technologies (Richardson and Lynes 2007). Not all building materials are considered environmentally friendly (Akadiri et al. 2012). A substance must be renewable, reusable, or recyclable to be considered sustainable, and the same can be said about innovations or technology. Another feature of a sustainable material or technology is its ability to improve health and social well-being. Sustainable resources, which are required to make a building truly green, are in short supply, posing a threat to sustainable construction practices (Choguill 2008; Shi et al. 2016). Summary of some of the important studies on the role of resources towards sustainable construction is presented in Table 4.

**Table 4:** Impacts of resources to barriers of SC

Factors	Sources	Methodology
Sustainable resources and technology limitations	Richardson and Lynes (2007)	Interviews
Limited supply of resources and materials	Choguill (2008); Shi et al. (2016)	Literature review, semi-structured interviews

### Conceptual framework

**Fig. 3:**
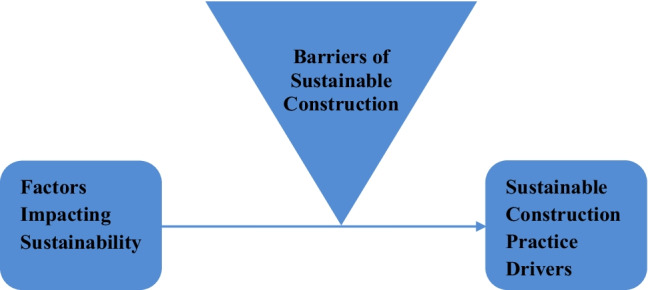
Conceptual framework of study

A conceptual model drawn from the aforementioned literature, consisting important factors was developed to test and provide important inferences in this study. Based on the literature, it is clearly mentioned in the Fig. 3 that different factors impacting sustainability help the sustainable construction practices. However, certain barriers play reverse role, not only in the sustainable construction practices, but also diminishing the relationship in between factors impacting sustainability and sustainable construction practices. A moderating role of barrier of sustainable construction in between factors impacting sustainability and sustainable construction practices is highlighted in Fig. 3.

The details of the conceptual model are highlighted in Fig. 3.

## Methodology

**Fig. 4:**
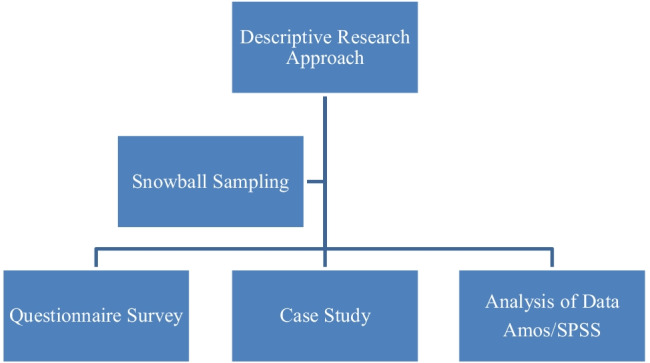
Research methodology structure

Research methodology structure of this study is presented in the Fig. 4.

### Research motive

This study is motivated the quest of the research to gain insight of the research questions through mix-method research by collecting and analysing responses to the questionnaire and survey, and then presenting relevant case study. The findings of the data collection will then help develop a model of framework for how factors and barriers of sustainable construction impact the practices of sustainable construction in UK. A gap exists in research on how sustainable construction practices is being driven by these variables.

### Research methods

This study aims at identifying and analysing the factors and barriers of sustainable construction from a managerial perspective, and therefore adopts the mixed-method research approach in collecting data to establish association or causal relationships between variables. Quantitative method is a systematic empirical investigation of quantitative properties and phenomenon. Whereas, for the qualitative data collection a case study about the Kier Group plc was analysed to back up the quantitative findings.

### Quantitative method

#### Sampling and data Collection

Given the large size of the target population, a decision was made to consider respondents with relevant years of experience in the UK sustainable construction industry. Also, the researcher finds it unrealistic to draw up sample frame as a result this research has no sample frame. However, sample selection will be criteria based, such that only samples which meet this criterion will be considered.

**Fig. 5:**
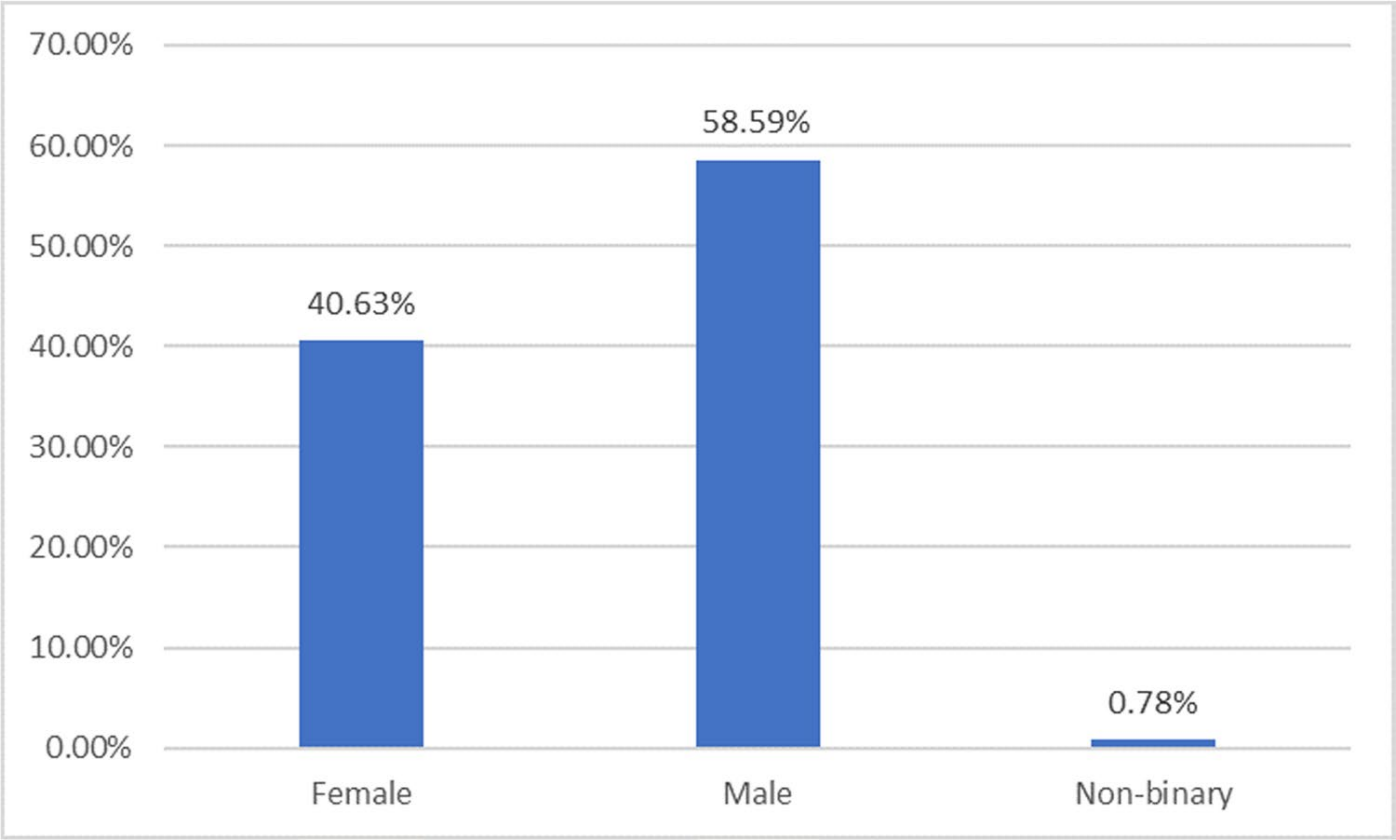
Gender of respondents

**Fig. 6:**
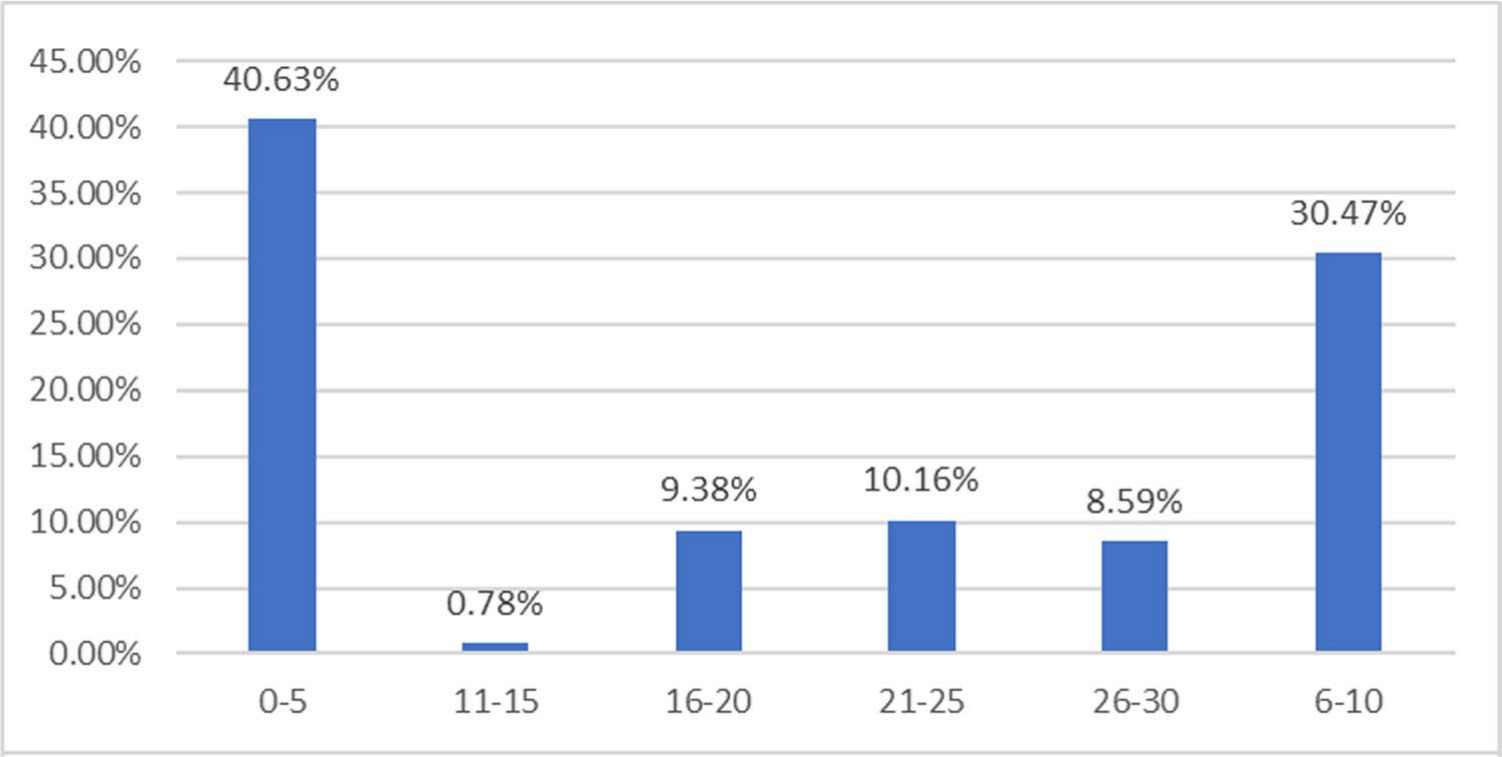
Years of work experience in UK construction industry

**Fig. 7:**
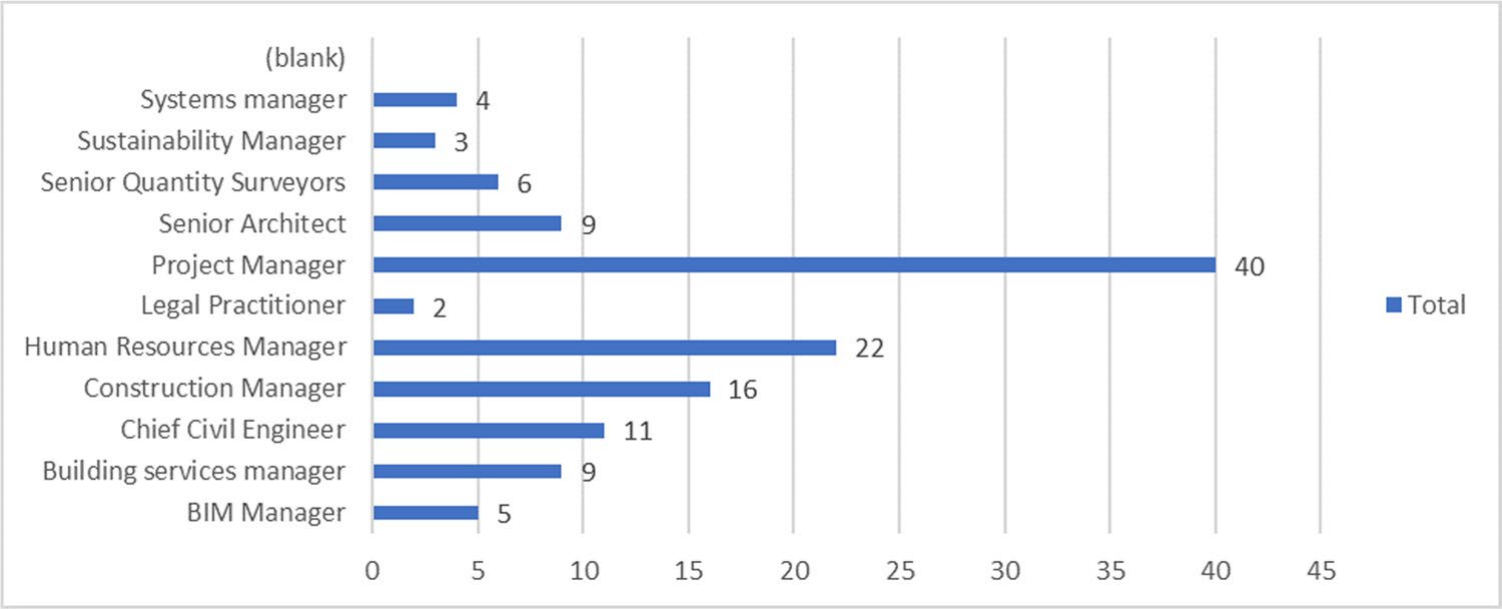
Managerial position of respondents

**Fig. 8:**
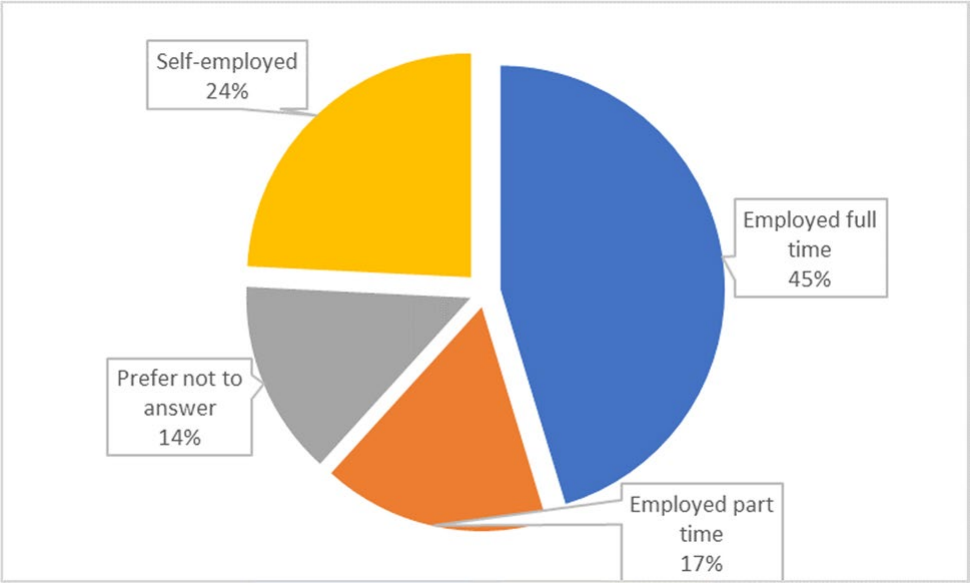
Employment status

**Fig. 9:**
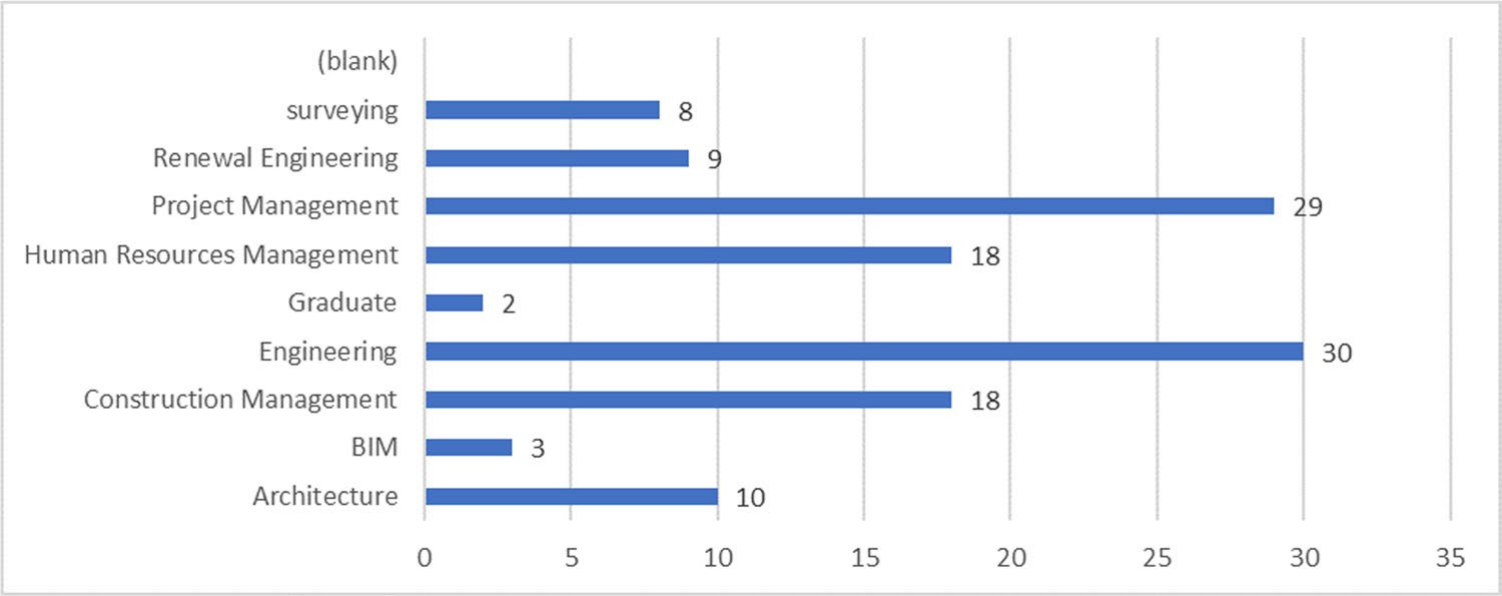
Educational background of respondents

Also, snowball sampling was used since the intended targets are industry managers with a considerable year of experience in such roles. Hence, only managers were administered with online questionnaire through emails and LinkedIn. Data for this study were collected via the use of a well structure questionnaire developed using JotForm. To administer the questionnaire, URL generated was sent via e-mail to the target respondents for completion of the online survey. The demographic details of the respondents are presented in Figs. 5 to 9.

Figure 5 depicts the gender of the respondents in this study across the different groups of respondents. Most of the respondents were male and female, accounting for around 41% and 59% of the responses, respectively, according to the data. Non-binary people make up less than 1% of the population.

The respondents’ years of experience in the UK construction industry are shown in Fig. 6. Approximately 41% of respondents have 0–5 years of experience, while 30% have 6–10 years of experience. While 9% of respondents have 16–20 years of experience and 26–30 years of experience, respectively, 10% have 21–25 years of experience and less than 1% have 11–15 years of experience. Our data indicates that the bulk of the respondents have a significant amount of experience working on building projects in the UK, and their replies will be extremely useful to this study.

Figure 7 depicts the respondents’ involvement in sustainable construction initiatives in the UK. Project managers account for 40% of those who responded, followed by 22% of human resource managers, 16% of construction managers and 11% of civil engineers. This implied that the responders in the study have significant clout in the UK construction industry.

The respondents’ occupational status is depicted in Fig. 8. The bulk of the respondents are employed, according to the data, accounting for around 86% of the total. Because they are currently employed in the business, the polled respondents can supply reliable and sufficient information.

The educational backgrounds of the respondents are shown in Fig. 9. The respondents have appropriate educational backgrounds that may be required in the construction business, such as project management, engineering, construction management, architecture, and renewal engineering.

#### Data screening

At the end of three weeks of data collection, 128 completed questionnaires were returned out of 180 that were distributed, representing a reasonably high response rate of 71.11%. The data was then collected through Jot Tables and then formatted within Microsoft Excel to create graphs to visualise the survey results. No missing values were observed in the data gathered from the survey respondents. Data obtained in section B to section D of the questionnaire were imported to MS Excel IBM SPSS Statistic 23.0 where different types of analysis took place. Missing responses were completed in the software.

#### Questionnaire development

A survey questionnaire was developed from information gathered through literature review of studies by authors like Pham et al. (2020), Tokbolat et al. (2020), AlSanad (2015), Abidin (2010), Abidin and Powmya (2014), Safinia et al. (2017) and Saleh and Alalouch (2015) which provided background information on research objectives. The questionnaire was designed in 5 sections.

i. Section A: Aimed at collecting respondents’ personal profile. A total of five questions are in this section. The questions were developed to allow the researcher have background knowledge of the respondents.
ii. Section B: Contains a list of 24 factors of sustainable construction previously identified from literatures of AlSanad (2015) and Safinia et al. (2017). The list was presented to the respondents to get their level of agreement with these factors of sustainable construction practice on a Likert scale of 1 to 5
iii. Section C: Contains a list of 13 barriers of sustainable construction previously identified from literatures of Pham et al. (2020), Tokbolat et al. (2020), AlSanad (2015) and Saleh and Alalouch (2015). The list was presented to the respondent to get their level of agreement with these barriers of sustainable construction practice on a Likert scale of 1 to 5
iv. Section D: Contains a list of 6 driving forces of sustainable construction previously identified from literatures of Tokbolat et al. (2020), Abidin (2010) and Abidin and Powmya (2014). The list was presented to the respondent to get their level of agreement with these drivers of sustainable construction practice on a Likert scale of 1 to 5.

#### Reliability and validity tests

Cronbach’s alpha coefficient test was used to analyse the internal consistency of the research instrument's numerous constructs to determine its reliability. Any value of Cronbach’s alpha is acceptable if it is over 0.6 (Ursachi et al. 2015), so the data collected for this purpose is found to be reliable to test in this study.

From Table 5, the reliability of the research instrument was found to be satisfactory. Some of the researchers from whose works the validity of this research is measured and confirmed to fit appropriately for what it is being used to measure are Pham et al. (2020), Tokbolat et al. (2020), AlSanad (2015), Abidin (2010), Abidin and Powmya (2014), Safinia et al. (2017) and Saleh and Alalouch (2015).

**Table 5:** Reliability of research instruments

Variable	Cronbach’s alpha	Cronbach’s alpha based on standardised items	*N* of items
Factors of sustainable construction	0.847	0.847	24
Barriers of sustainable construction	0.781	0.781	13
Sustainable construction practices	0.770	0.769	6

### Qualitative method

Qualitative method for this research was based on the analysis of the case study of the Kier Group plc. Kier Group plc is a UK-based construction, services and property group which is actively engaged in building and civil engineering related businesses. The case of Kier Group plc chosen as this group is actively engaged with multiple sustainable construction projects. Kier Group plc follow the main two factors of sustainability, which are environmental responsibility along social responsibility. Kier Group Plc maintains their legal environment as well as a social responsibility that helps them develop performance build a strong and eco-friendly environment as well as community and also help to generate strong and sustainable profits (Kier Group plc. 2021). Moreover, the descriptions of this case’s sustainable practices would support the quantitative findings in this research.

## Data analysis

The obtained data is analysed in this section so that the researcher can discuss the findings and form conclusions about the study issues. To draw conclusions from the data, statistical tests such as mean analysis, relative importance index (RII) and bivariate correlation analysis were used.

### Quantitative analysis

Quantitative analysis was conducted by using the mean analysis, RII, bivariate correlation and hierarchical regression analysis. The mean analysis, bivariate correlation and hierarchical regression analysis were conducted by using the SPSS software; however, RII was performed manually by MS excel.

The RII was conducted to understand the ranking of the factor effecting sustainability and barriers in the sustainable construction practices. The higher the RII value, the greater the significance of the particular factors affecting sustainability or barrier (Gebrehiwet and Luo 2017). The RII was conducted by using the following equation:

$$\mathrm{RII}=\sum \left(\mathrm{Wi}\right)\left(\mathrm{Fi}\right)/\mathrm{A}*\mathrm{N}=1\left(\mathrm{F}1\right)+2\left(\mathrm{F}2\right)+3\left(\mathrm{F}3\right)+4\left(\mathrm{F}4\right)+5\left(\mathrm{F}5\right)/5\left(\mathrm{N}\right)$$

The RII was calculated using the following Waziri and Vanduhe (2013) guide for calculating RII values:

1. 0.76 and above Most significant
2. 0.67–0.75 Significant
3. 0.45–0.66 Less significant
4. 0.44 below Not Significant

Using the mentioned equation, the RII values for barriers to sustainability and barriers of sustainable construction are provided in Tables 6 and 7.

**Table 6:** RII analysis of the driving forces of sustainable construction

Variables	N	1	2	3	4	5	Mean	RII	Rank
Sustainable construction designs	128	0	0	8	81	39	4.21	0.85	1st
Principles of sustainable development	128	0	0	10	81	37	4.11	0.84	2nd
Interest in sustainability	128	0	0	13	77	38	4.24	0.84	2nd
Technological advancements and innovations	128	0	0	16	71	41	4.20	0.84	2nd
Performance measurement Systems	128	0	0	16	76	36	4.20	0.83	3rd
Greenhouse features	128	0	0	14	86	28	4.16	0.82	4th

**Table 7:** RII analysis of barriers of sustainable construction practices

Variables	N	1	2	3	4	5	Mean	RII	Rank
Economic-related barriers	Mean: 4.11								
Low understanding of economic benefits	128	0	0	18	81	29	4.09	0.830159	5th
Excessive concentration on price	128	0	0	19	81	38	4.15	0.906349	1st
Potential extension of schedule	128	0	0	24	80	24	4.00	0.812698	6th
Economic conditions	128	0	0	13	77	38	4.20	0.852381	2nd
Risk associated with implementation of new practices	128	0	0	18	79	31	4.10	0.833333	4th
Sustainable construction is expensive	128	0	0	24	64	40	4.13	0.838095	3rd
Government-related barriers	Mean: 4.15								
Lack of government incentives	128	0	0	15	76	37	4.17	0.847619	2nd
Unclear laws and regulations from government	128	0	0	13	76	39	4.20	0.853968	1st
No existing rule in the UK to adopt sustainable construction	128	0	0	30	59	39	4.07	0.826984	3rd
Resource-related barriers	Mean: 4.22								
Limited sustainable materials and technologies	128	0	0	11	80	37	5	0.853968	2nd
Lack of human resource	128	0	0	11	75	42	5	0.861905	1st
Culture related barriers	Mean: 4.16								
Maintaining the current practice and resisting the change towards sustainability	128	0	0	10	80	38	5	0.857143	1st
Low implementation level of sustainable practices	128	0	0	17	80	31	5	0.834921	2nd

#### RII analysis of drivers of sustainable construction practices

The survey respondents were asked to rank the various driving forces of sustainable construction techniques in order of importance. A 5-point Likert scale ranging from low to high priority was offered to the respondents. Each variable’s RII was calculated.

The resulting analysis are shown in Table 6.

Each variable has a significant significance value of 0.82 and above, as seen in the Table 6. The significant difference between the primary variables is proved to be 0.1, confirming that a 99% confidential limit exists and demonstrating that for a sustainable structure to exist, variable (control) from the above must link the dependency of barriers and activities. As argued by academics, what the Table 6 reveals aligns and coincides with their findings from relevant literatures evaluated on the driving forces of sustainable construction (Whang and Kim 2015; Ahn et al. 2013; Choguill 2008; Gunatilake and Perera 2018). These driving elements are enhancers of sustainable construction practises in the UK by the sampled respondents in the management cadre.

#### RII analysis for barriers to sustainable construction

Participants in the poll were asked to rate how much they agreed with the following barriers to using sustainable construction practises. The respondents were given a 5-point Likert scale ranging from strongly disagree to strongly agree. The average response to each obstacle was determined using the mean and RII. Table 7 summarises the findings of the study.

Table 7 explains the RII analysis of barriers of sustainable construction from the minimum and maximum point of analysis. From the result above, ‘excessive concentration on price’ barriers pull a maximum RII value of 0.906349, ahead of other barriers. The implication here is that highest prices of the sustainable materials and technologies hinders or create significant barriers to sustainable construction. The prices are the key factor in deciding about any project, thus it forms a direct relationship with sustainable construction. ‘Lack of human resource’ creates a second major barriers to sustainable construction with its RII value of 0.861905. ‘Limited human resources’ create direct relationship to barriers of sustainable construction. Limited capacity of the efficiency of human resource creates how productivity of effort to match significant actions of sustainable construction. It was also observed that the ‘resource-related barriers’ have the highest position among other barrier categories with its mean value of 4.22, whereas the ‘economic related barriers’ found to be the least significant with mean value of 4.11.

#### Bivariate correlation analysis

According to Table 8, there is a strong, positive, and substantial relationship between sustainable construction factors, barriers and drivers.

**Table 8:** Bivariate correlation

			Barriers	Factors	Sustainable construction practices
Spearman’s rho	Barriers	Correlation Coefficient	1.000	0.671^**^	0.462^**^
		Sig. (2-tailed)		0.000	0.000
		N	127	127	127
	Factors	Correlation Coefficient	0.671^**^	1.000	0.656^**^
		Sig. (2-tailed)	0.000		0.000
		N	127	127	127
	Sustainable construction practice drivers	Correlation Coefficient	0.462^**^	0.656^**^	1.000
		Sig. (2-tailed)	0.000	0.000	
		N	127	127	127

#### Multiple hierarchical regression analysis

Table 9 presents the moderating effects of barriers in between the impact of sustainability factors on sustainable construction practices.

**Table 9:** Hierarchical regression of barrier impact on sustainability factors

Variable entered	Step 1		Step 2		Step 3	
	Standardised coefficients	*p* value	Standardised coefficients	*p* value	Standardised coefficients	*p* value
	Beta		Beta		Beta	
Stakeholders’ factors	0.121	0.009	0.069	0.434	0.370	0.673
Project management factors	0.473	0.000	0.438	0.000	-0.127	0.898
Technological factors	0.263	0.001	0.258	0.001	0.654	0.532
Barriers			0.101	0.302	0.093	0.873
Stakeholders’ factors × barriers					-0.503	0.741
Project management factors × barriers					0.975	0.568
Technological factors × barriers					-0.559	0.712
*F* value	40.056		30.329		17.007	
*F* change	40.056		1.075		0.12	
*R* square	0.494		0.499		0.5	

Result of the hierarchical regression analysis shows that there is a significant direct effect between the factors of sustainability (stakeholder, project management and technological factors) and the driving forces of sustainable construction practices (see Table 9). No significant effects were observed via the moderation variable (barriers of sustainable construction). Therefore, the model of moderation test was not accepted as the insignificant values are observed, as highlighted in Table 9.

### Qualitative analysis

In this section, themes have been developed based on aim, objective as well as questions so that the outcome of this research may not deviate from its ultimate goals.

#### Qualitative results based on the case study findings on Kier Group plc

**Fig. 10:**
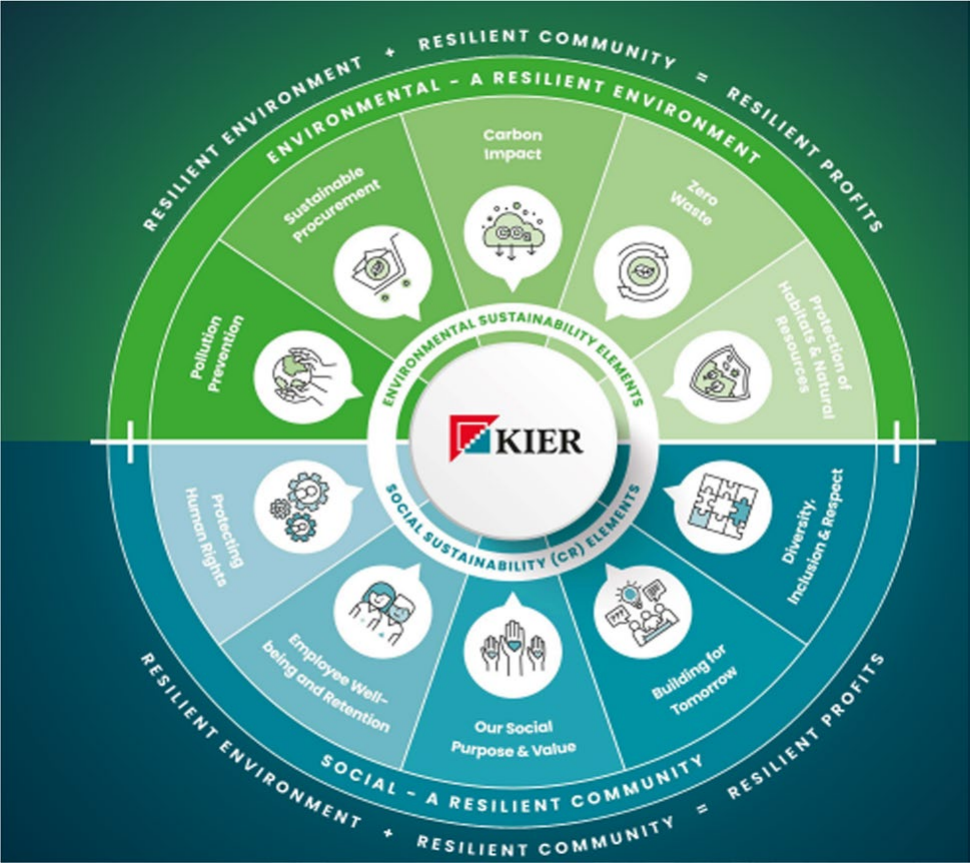
Sustainability framework of Kier Group plc ( Source: Adapted from Kier Group plc. 2021, p.7)

Kier Group plc believes that ‘green is a trend, sustainability is a mindset’. The main three features that they follow to make their business sustainable are a strong environment, a strong community that involves workforce, consumers as well as suppliers, and finally strong profit. The main two factors of sustainability are environmental responsibility along social responsibility. Kier Group plc maintains their legal environment as well as a social responsibility that helps them develop performance build a strong and eco-friendly environment as well as community and also help to generate strong and sustainable profits (Kier Group plc. 2021). Kier Group Plc encourages their employees and workers to adopt this mindset as well as they try to maintain their business by following three main features such as community, environment, and sustain profit to operate their sustainable business. For building a sustainable business they also focused on two more factors that are environmental sustainability as well as social sustainability. Kier Group plc implements ten actions in these two areas to identify more important things in the environment along with social concerns. These ten pillars mainly focus on where they can achieve the greatest strength by their operation. Their new target regarding achieving sustainability in their business is net-zero carbon across their supply chain as well as operations by 2045 and minimising waste by 2035. Kier Group plc developed different strategies to meet its specific target. For maintaining better governance, they create a sustainable leadership forum. Reason behind applying this action is to ensure sustainable actions and improve their business decisions (Kier Group plc. 2021). Figure 10 highlights the sustainability framework of Kier Group plc.

#### Different intuitive taken by Kier Group plc to increase environmental sustainability

Kier Group plc effectively supports and trains as well as audits their project to reduce the risk of pollution. They also developed modern commercial KPIs to measure their pollution along with the impact of this pollution, try to build a project which reduces pollution incidents and also implement different innovative technologies and best practices for reducing population.

Kier Group plc manages their resources by improving knowledge as well as the use of sustainable materials to use their full potentiality of materials and reduce waste. Enhance investment in their sustainable materials, generating values for consumers by use of sustainable materials based on their life cycle as well as actively monitor and promote materials that fit with the changing economy. They implement different strategies to reduce their carbon emission as well as to adapt and increase the ENCORD protocol (Kier Group plc. 2021).

Kier Group plc reduces their waste by implementing proper direction and commercial KPIs for reducing direct as well as indirect waste costs. Implementing zero-waste avoidance principles to build sustainability as well as recycle that waste for their further production process. They also build some economic principles to resign as well as manage their wastage process and enhance the percentage of recyclable waste to building materials (Kier Group plc. 2021). They also take very important intuitive steps to protect the environment and resources by adopting various actions and comprehensive tools to measure biodiversity, improve natural commitment principles, and develop plans for reducing water wastage as well as recycling water for further production processes.

#### Different intuitive taken by Kier Group plc to increase social sustainability

Kier Group plc builds sustainability in supply chain management as well as invested in online programs and training regarding sustainability to develop the supply chain. Review, reduce as well as renew and rebalance are the most important factors to maintain social sustainability (Kier Group plc. 2021). They also contribute to building sustainable education, sustainable employment and supporting small businesses and sectors, etc. Building a sustainable environment creates wealth for the nation, increasing employment as well as career opportunities. They arrange different events, a campaign to increase the awareness about sustainability between peoples and society. Kier Group plc increases social sustainability by creating employment in the UK, which played a very important role in growth of the economy and also supports small industries along with sectors to adopt to increase social sustainability. Building good communication with consumers, suppliers, and supply chain management helps them to understand climate change as well as gives them an edge to prepare themselves for upcoming changes (Kier Group plc. 2021).

## Discussion

The purpose of this section is to align the study’s findings with the study’s goal and objectives, as well as the research questions that motivated it. To that end, the research findings will be discussed under the following sub-topics: driving forces of sustainable construction, barriers of sustainable construction practises, analysis of actions of sustainable construction practises, and relationship between barriers and actions of sustainable construction practises.

### Sustainable construction drivers

The leading driver of sustainable construction, according to this survey, is sustainable construction designs. This indicates that most managers feel that a sustainable construction design may appeal to both construction managers and clients. This finding is in line with Hwang and Tan (2012) who opined that most experts agree that sustainability in building operations should begin with the planning stage and be represented in the design. This finding is also backed up by Gunatilake and Liyanage (2010) who noted that sustainable construction design is the first and most important step toward taking on long-term responsibility for the building sector.

Sustainable construction design was closely followed by principles of sustainable development, interest in sustainability, technological advancements, and innovations. These three variables share the same RII of 0.84. This implies that managers believe that principles of sustainable development, interest in sustainability, technological advancements and innovations are all most significant drivers of sustainable construction. Next in line is performance measurement systems which is also closely followed by green-house features. Using Waziri and Vanduhe (2013) guide for rating RII values, it has been established that all the variables considered as drivers of sustainable construction are most significant because none of the considered variable is lower than 0.76 RII. Hence, sustainable construction design, principles of sustainable development, interest in sustainability, technological advancements and innovations, performance measurement systems and green house features are all important drivers of sustainable construction. Therefore, they should be taken into consideration while embarking on a sustainable construction project.

### Barriers of sustainable construction

This study found out that among the economic associated barriers, ‘excessive concentration on price’ has the highest RII value of 0.906349 indicating a high degree of agreement among mangers who are involved in sustainable construction practices in the UK. This means that the cost of sustainable construction is a barrier to its acceptance by clients. However, the benefits of sustainable construction outweigh the cost. This study also rates ‘economic condition’ of a country next to the ‘excessive concentration on price’. The ‘economic conditions’ was observed to be the second important barrier to sustainable construction in the UK with its RII value of 0.852381. It is therefore established that the economic condition of a country at the time determines the acceptance of sustainable construction. Closely related to the issue of price is the belief that sustainable construction practice is expensive with third position and having the RII value of the 0.838095. This line of thought runs through Häkkinen and Belloni (2011), Sodagar and Fieldson (2008), and Zhou and Lowe (2003). All the studies are of the view that the cost of sustainable construction practice determines its acceptance among clients.

There is also the issue of risk factors that are associated with stating up a new idea. From this study, it was found that many managers believe that clients are usually worried about the risks that are likely to come along with the adoption of sustainable construction practice. Next to this is the fact that several people do not understand the enormous economic benefits that come with sustainable construction. This finding gives credence to Daniel et al. (2018) who found out that a lack of the understanding of the economic benefits of sustainable construction is a major barrier to sustainable construction practices. The least considered barrier by the respondents surveyed is potential extension of schedule with its RII value of 0.812698. This does not in any way undermine the fact that clients are also worried about the possible extension of date scheduled for the completion of sustainable construction projects. According to the preceding, ‘excessive concentration on price’ and ‘economic condition’ of a country have a detrimental impact on sustainable construction in the UK.

This study found out that among the government-related barriers, ‘unclear rules and regulations from government’ had the highest RII value of 0.853968. This means that a good number of managers are of the opinion that unclear rules by the government of the UK on sustainable construction has so far been a major barrier to the acceptance of sustainable construction practices among clients and construction experts. The mangers also share the view that a ‘lack of incentive from the government’ to support sustainable construction practice is an impediment to the progress of the practice in the UK with RII value of 0.847619. Another government factor that stands as a barrier is that there is ‘no existing rule in the UK to adopt sustainable construction’ with RII value of 0.826984. Rules on sustainable construction are important and can stand as a barrier in their absence for the sustainable practices in the industry (Heeres et al. 2004; Serpell et al. 2013; Samari et al. 2013). Thus, it has been confirmed that government unwillingness has a negative role on sustainable construction in the UK.

For resource-related barriers, this study found that ‘lack of human resources’ and ‘limited sustainable materials and technologies’ are the important barriers to sustainable construction with RII values of 0.861905 and 0.853968, respectively. Hence it is emphatically stated that the ample lack of human resource and limited sustainable material as well as limited technological available equipment are barriers of sustainable construction in the UK. These findings give credence to Richardson and Lynes (2007) who noted that a lack of sustainable materials and technologies. These finding are also in line with Choguill (2008) and Shi et al. (2016) who equally noted that the sustainable resources are in short supply. Thus, it is confirmed that managers in the UK agree that insufficient resources have negative impact on sustainable construction.

This study found out that managers in the UK agree that cultural related barriers have negative impact on sustainable construction. According to the survey carried out in this study, both the ‘maintaining the current practice and resisting the change towards sustainability’ and ‘Low implementation level of sustainable practices’ are found to be the major barriers to the sustainable construction practices in the UK with RII values of 0.857143 and 0.834921, respectively. This shows that cultural beliefs are the most outstanding barriers to the adoption of sustainable construction practices in the UK. Williams and Dair (2007) and Ametepey et al. (2015) share the view that the culture of accepting or neglecting sustainable is a barrier and their opinion is in sync with the findings of this study. Thus, cultural resistances have negative impact on sustainable construction in the UK.

### Relationship between barriers and actions of sustainable construction

This study showed a strong, positive, and significant link between sustainable building activities, barriers and drives. The hierarchical regression analysis results show that the sustainability determinants (stakeholders, project management, and technological variables) have a significant direct influence on the driving forces of sustainable construction practises. The moderation variable had no effect on the outcomes (barriers of sustainable construction). As a result, it is possible to conclude that this study does not support the moderation test.

According to the findings, actors and barriers have a substantial impact on sustainable construction practises in the UK. To put it another way, the future of sustainable construction in the UK is totally dependent on how well the drivers, actors and impediments to sustainable construction in the UK are managed.

## Conclusion and recommendations

This study has provided a strong base from literature review to analysis and then discussion on the impediments and factors impacts on the sustainability of the construction projects in the UK. During the review of literatures many factors were identified ranging from culture, socio-economic, environment, stakeholders and project management practices. From literatures, there is little works done on the perspectives of clients on what are the triggers and impediments to sustainable construction in the UK. Several variables inspire construction organisations with the desire to achieve sustainability; nonetheless, construction design has been highlighted as the most major driver of sustainable building. Regardless of this, every other driver of sustainable construction according to this research was considered significant. The economy of the UK can be a barrier to sustainable construction practice because most managers agree that the cost of sustainable construction practice is a barrier. Other barriers include government, resources and cultural related barriers.

A mixed-method research approach was utilised to collect the data to perform the analysis techniques. The quantitative data was collected through questionnaire survey, for this purpose a snowball sampling was applied to collect the questionnaire responses from the 128 managerial roles working in UK construction industry. A case study of Kier Group plc was chosen to understand the sustainable construction practices in the UK construction industry. In order to perform quantitative analysis, the mean, correlation, RII and hierarchical regression analysis techniques were utilised. The RII analysis discovered that sustainable construction designs is a top drivers of sustainable construction practices, whereas excessive concentration on price is found as the top impediment of sustainable construction practices. It was also shown by the hierarchical regression analysis that stakeholders factors, project management factors and technological factors significantly impact to sustainable construction practice. However, surprisingly the role of barriers was not observed in the sustainable construction industrial practices of the UK. Future research on identifying barriers and actions of sustainable construction from industry executives should look at comparing between two developed countries as well as between a developing country and developed country to draw any similarity and differences in opinions.

## Data Availability

Data generated or analysed during the study are available from the corresponding author on reasonable request.

## Abbreviations

UK: United Kingdom
RII: Relevant importance index
Plc: Public limited company
CIB: Conseil International du Bâtiment
BIM: Building Information Modeling
SC: Sustainable construction
IBM: International Business Machines Corporation
SPSS: Statistical Package for the Social Sciences
MS: Microsoft
N: Number
KPI: Key performance indicator
ENCORD: European Network of Construction Companies for Research and Development
